# Hyperschematia after right brain damage: a meaningful entity?

**DOI:** 10.3389/fnhum.2014.00008

**Published:** 2014-01-28

**Authors:** Gilles Rode, Roberta Ronchi, Patrice Revol, Yves Rossetti, Sophie Jacquin-Courtois, Irene Rossi, Giuseppe Vallar

**Affiliations:** ^1^INSERM U1028, CNRS UMR5292, Centre de Recherche en Neurosciences de Lyon, Equipe ImpActBron, France; ^2^Service de Médecine Physique et Réadaptation, Hôpital Henry Gabrielle, Hospices Civils de LyonSaint Genis Laval, France; ^3^Laboratory of Cognitive Neuroscience, School of Life Sciences, Brain Mind Institute, Ecole Polytechnique Fédérale de LausanneLausanne, Switzerland; ^4^Dipartimento di Psicologia, Università degli studi di Milano-BicoccaMilano, Italy; ^5^Neuropsychological Laboratory, IRCCS Istituto Auxologico ItalianoMilano, Italy

**Keywords:** hyperschematia, extra-personal space, representation, anisometry

## Abstract

In recent years we reported three right-brain-damaged patients, who exhibited a left-sided disprortionate expansion of drawings, both by copying and from memory, contralateral to the side of the hemispheric lesion (Neurology, 67: 1801, 2006, Neurocase 14: 369, 2008). We proposed the term “hyperschematia” for such an expansion, with reference to an interpretation in terms of a lateral leftward distortion of the representation of extra-personal space, with a leftward anisometric expansion (relaxation) of the spatial medium. The symptom-complex shown by right-brain-damaged patients with “hyperschematia” includes: (1) a disproportionate leftward expansion of drawings (with possible addition of details), by copy and from memory (also in clay modeling, in one patient); (2) an overestimation of left lateral extent, when a leftward movement is required, associated in some patients with a perceptual underestimation; (3) unawareness of the disorder; (4) no unilateral spatial neglect. In most right-brain-damaged patients, left “hyperschematia” involves extra-personal space. In one patient the deficit was confined to a body part (left half-face: personal “hyperschematia”). The neural underpinnings of the disorder include damage to the fronto-temporo-parietal cortices, and subcortical structures in the right cerebral hemisphere, in the vascular territory of the middle cerebral artery. Here, four novel additional patients are reported. Finally, “hypeschematia” is reconsidered, in its clinical components, the underlying pathological mechanisms, as well as its neural underpinnings.

## Introduction

Productive—or positive—symptoms can be defined as behavioral manifestations by the generation of acts, or verbal reports, and more generally, additions to “normal” experiences (Malaspina et al., 2013). Productive symptoms may be contrasted with defective—or negative—symptoms, whose hallmark is the absence of a behavior (act, response), appropriate to the situation. The productive/defective distinction has been used to characterize symptoms and signs of a number of neurological, neuropsychological, and psychiatric disorders. In general, productive manifestations may reflect a dysfunctional, distorted, rather than merely “defective” representation of reality (Jackson, 1879, 1932; Sandson and Albert, 1984; Goldberg, 1986).

In the spatial domain, productive symptoms may be associated with right brain damage and the syndrome of spatial unilateral neglect (Vallar, 2001). With reference to the different spatial frames (Vallar and Maravita, 2009), positive symptoms can involve personal space, such as in the case of somatoparaphrenia (Vallar and Ronchi, 2009, for review; Gerstmann, 1942), and near extra-personal/peri-personal spaces. In the latter, within hand-reach space, productive symptoms may be evidenced by tasks involving visuo-motor exploration or drawing, where patients may produce perseverations in target cancellation (Rusconi et al., 2002; Vallar et al., 2006), and drawing from memory or by copying a scene (Ronchi et al., 2009). This perseveration behavior mainly concerns the side of space ipsilateral to the side of the hemispheric lesion (ipsilesional), ranging from the repeated cancelling out of targets to the addition of new targets, as well as and more complex graphic productions.

Much less frequently, this behavior may be evidenced in the side of space contralateral to the side of the lesion (contralesional). An early description was made by the British neurologist MacDonald Critchley in his seminal book “The parietal lobes” (Critchley, 1953). Critchley reported three patients with a right-hemispheric brain tumor, who exhibited a left-sided expansion of the drawing, contralateral to the side of the lesion, when required to draw a daisy from memory (see also Guariglia et al., 1998).

A few years ago, we reported three right-brain-damaged patients, who exhibited productive, rather than defective, responses, in the left side of near extra-personal space, characterized by systematic left-sided expansion of drawings, both by copying and from memory, and addition of left-sided details to the drawn object (Rode et al., 2006, 2008). In one patient, this leftward expansion in 2-D drawing involved also clay modeling, in a 3-D visuo-constructive task (Rode et al., 2008). This pathological behavior persists when patients are blindfolded during the task, suggesting that the deficit is not confined to the visual modality. With reference to these productive manifestations, Rode et al. (2006) proposed the term “Hyperschematia” (HS), as a tribute to the French otolaryngologist Bonnier (1905; Vallar and Rode, 2009). Bonnier described patients suffering from a peripheral vestibular disorder, who reported the phenomenal experience of body parts as disproportionately large, as if 3D-expanded. HS is likely to reflect a perceptual, rather than premotor, size distortion. Indeed, when required to make a perceptual judgment about the lateral extent of two rectangles, all reported HS patients underestimate the left-sided stimulus. In a visuo-motor task requiring the reproduction of horizontal extent (line extension task, using the right unaffected hand), patients exhibit a leftward “overextension,” namely patients produce through a leftward movement a horizontal segment longer than the model. This “productive” (in the line extension task, since the response is a segment longer, rather than shorter, than required) behavior occurs when the direction of the movement of the right upper arm is leftwards, contralateral to the side of the right hemispheric lesion. Conversely, when the extension is performed rightwards the patients' reproduction of the lateral extent of the line is within the normal range. This disproportionate leftward extension may reflect a distorted representation of extra-personal space, or of the “spatial medium” [discussion in Vallar (2009)]. Paradoxical disproportionate leftward extensions by right-brain-damaged patients with left spatial neglect in variants of the line bisection task have been repeatedly reported [Bisiach et al. (1994) setting the endpoints of a line, given its midpoint; Gallace et al. (2008), for related evidence; see also converging findings evidence in Lenggenhager et al. (2012), using moving stimuli]. In these studies, disproportionate leftward extensions, however, were found in patients with left neglect, and with different tasks. A group study (Bisiach et al., 1998) performed in a large series of right-brain-damaged patients, with and without left spatial neglect has shown that left contralesional disproportionate overextension in a line extension task is a phenomenon, that occurs in right-brain-damaged patients both with and without left neglect in bisection and cancellation. This suggests that the two disorders: left overextension of the line (here discussed as a positive sign), and left neglect (omission in target cancellation and rightward error in line bisection) negative sign are likely to be independent, though frequently co-occurring phenomena. This conclusion is in line with the original findings by Rode et al. (2006) from HS patients without left neglect, who show left overextension.

The deficit described by Rode et al. (2006, 2008), may involve a disproportionate pathological leftward “relaxation” of the spatial medium, so that the left side of symmetrical objects is perceived by left HS patients as smaller, and compensatorily expanded leftwards in sensory (with and without visual control) -motor tasks. This behavior, on the one hand may be taken as an indication of the patients' lack of awareness of the perceptual disorder. On the other hand, it may reflect a preference for symmetry, stemming from basic perceptual mechanisms, that possibly initially arose during prehistory (Hodgson, 2011). A broadly similar pattern of HS, but ipsilateral to a right frontal lesion, was described by Saj et al. (2011) in a patient with no evidence of unilateral spatial neglect, and rightward ipsilesional expansion of drawings both from memory and by copy, with rightward perseveration, when drawing from memory: more petals of a flower on the right-hand side of the drawing; drawing of more sides of an unfolded cube on the right, actually resulting in an incorrect number of faces in the “exploded” cube (one more than required on the right, and possibly another half, left uncompleted). Other clinical reports of distortions of extra-personal and personal spaces, characterized by an underestimation (“hyposchematia”), rather than by an overestimation (“hyperschematia”) are on record. Two right-handed right-brain-damaged patients with no left spatial neglect reported the phenomenal experience that their face, and limbs, were disproportionately smaller on the left side. In drawing from memory, the left side of objects such as a butterfly, a house, and a daisy was disproportionately smaller, with added perseveration responses (i.e., more petals, repeated strokes), being unaware of the hyposchematic deficit in drawing (Kumral et al., 2012).

In this study, we report four novel right-brain-damaged patients who show a pattern of left-sided spatial distortion (“hyperschematia”), comparable to the one previously reported by Rode et al. (2006, 2008) in three patients. On the basis of this larger series of patients, as well as of observations reported by other investigators, since the original study by Rode et al. (2006), we reconsider the pattern of HS, and its putative dysfunctional mechanisms.

## Patients and control participants

Seven patients (P#1–P#7; two females, P#2 and P#5; six right-handed, one ambidextrous; mean age: 61.8 years, range 34–78; mean years of schooling: 10, range 8–18) participated in the study. All patients showed no history or evidence of previous neurologic and psychiatric disorders, or dementia. The patients' demographic and neurological features are summarized in Table 1: patients P#1–P#3 were previously described by Rode et al. (2006, 2008). Patients (P#4–P#7) are novel, for a total of seven patients.

**Table 1:** **Demographical and neurological features of seven right brain-damaged patients**.

**Patients**	**Age/sex**	**Laterality**	**Motor deficit**	**Somatosensory deficit**	**LHH**	**Line cancellation test omissions**	**Star cancellation test omissions**	**Etiology**
P#1	61/M	R	Severe	Severe	Absent	0	1^*^	Isch
P#2	45/F	R	Moderate	Absent	Absent	0	NA	Isch
P#3	73/H	R	Moderate	Moderate	Present	0	NA	Hem
P#4	57/H	R	Severe	Severe	Present	0	1^*^	Isch
P#5	79/F	R	Moderate	Moderate	Present	0	0	Isch
P#6	59/H	R	Severe	Severe	Present	0	0	Isch
P#7	50/H	A	Severe	Moderate	Present	1^**^	0	Isch

Visual fields were assessed by Goldmann perimetry. In the line cancellation test, patients had to crossed out 40 lines (Albert, 1973), In the star cancellation test, 56 targets (Wilson et al., 1987).

Abbreviations:R, Right-handed; A, Ambidextrous; LHH, Left homonymous hemianopia; Isch, Ischaemic; Hem, Hemorrhagic; NA, Not assessed.

* One omission in the center of the display.

** One omission in the rightmost part of the display.

**Figure 1:**
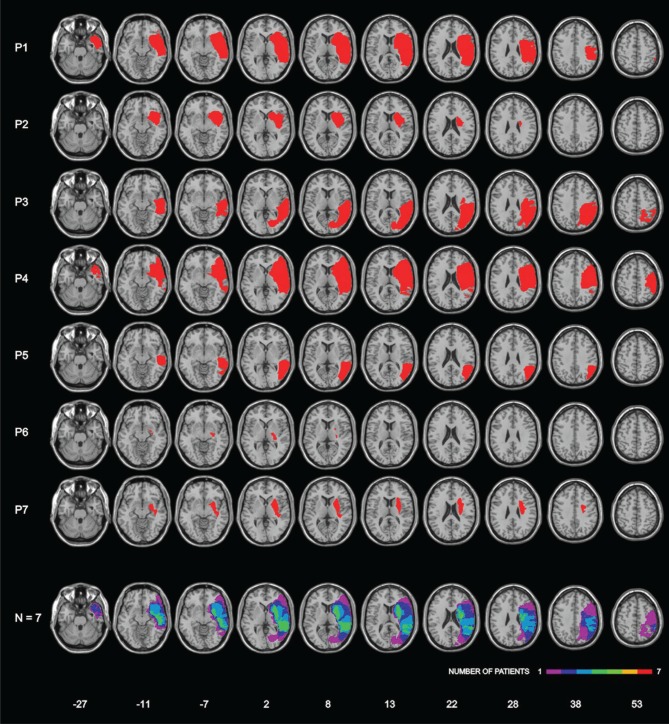
**Lesion localization in seven right-hemisphere-damaged patients with right-hemispheric lesions, and lesion overlap.** Bottom row: frequencies of overlapping lesions, from violet (*n* = 1) to red (*n* = 7). Montreal Neurological Institute (MNI) Z-coordinates of each transverse section are reported.

Lesions were assessed by CT or MRI. Ischemic lesions involved the vascular territory of the right middle cerebral artery in six patients; one patient (P3) had a hemorrhagic lesion in the temporo-parietal region. The patients' lesions were drawn on a standard MRI template with a 1-mm slice distance (voxels of 1 mm^3^) using MRIcro software (Rorden and Brett, 2000, www.mricro.com). This procedure required to adapt the standard template to each CT/MRI patient's orientation. Subsequently, each lesion was manually drawn on the correspondent adapted template and, finally, translated, in order to return to the parameters of the original MRI template. Lesion mapping was performed by Irene Rossi, who was naïve as to the patients' deficit, and checked by Roberta Ronchi and Giuseppe Vallar. Figure 1 shows the lesions of each patient, and the lesions' overlap of the seven patients. The maximum overlap (*n* = 6 patients) was in a small area in the white matter under the right temporal lobe; moreover, 5 out of 7 patients showed common lesions including the superior temporal cortex, the putamen and subcortical white matter under temporal and parietal (post-central) cortices.

All patients showed evidence of left spatial neglect at the bedside assessment, within one month post-stroke onset. At the time of the exam, two months after stroke onset in six patients (P#1, P#2, P#3, P#5, P#6, and P#7), five months in P#4, left neglect had recovered. All patients were examined in the same room of the Médecine physique et Réadaptation (Hôpital Henry Gabrielle, Lyon, France), by the same examiners (Gilles Rode, Patrice Revol, Sophie Jacquin-Courtois), and in the same conditions of light, and time of the day. No head and gaze deviation toward the side of the lesion was noted. Patients showed neither vestibular disorders (such as ataxia, vertigo, dizziness, nystagmus), nor symptoms of otholitic origin, such as room tilt, skew deviation and axial or limb latero-pulsion toward the right or the left side, in the acute stage post-stroke onset, and at the time of the exam. No auditory or visual extinction to double simultaneous stimulation was noted. All patients were aware of their motor and somatosensory deficits. They scored normally on line and star cancellation tests (Albert, 1973; Wilson et al., 1987), and showed no spatial neglect on tasks requiring drawing from memory or by copying objects (Grossi and Trojano, 2001), or a complex figure (Gainotti et al., 1972). Moreover, during all rehabilitative activities, as well as during the evaluations made by the occupational therapist at the hospital, all seven patients never showed signs of spatial neglect in the execution of attention-demanding ecological activities.

**Figure 2:**
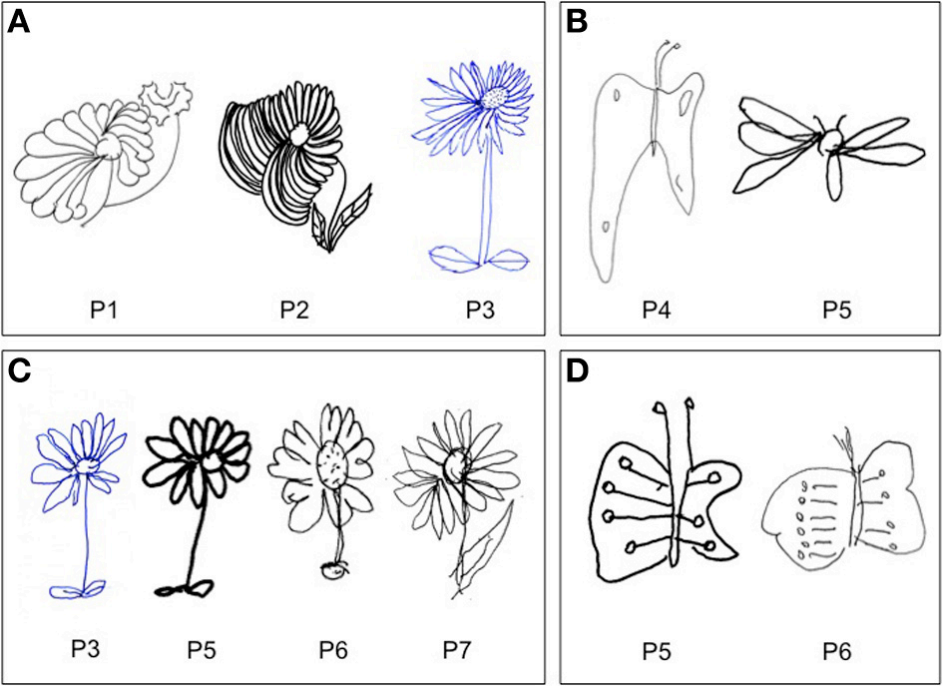
**Examples of drawing from memory (A, P#1, P#2, and P#3: daisy), (B, P#4 and P#5: butterfly) and by copy (C, P#3, P#5, P#6, and P#7: daisy), (D, P#5 and P#6: butterfly).** Patients showed a contralesional productive behavior, characterized by systematic left-sided expansion of drawings both by copying and from memory and addition of more left-sided details to the drawn object, contralateral to the side of the lesion.

**Figure 3:**
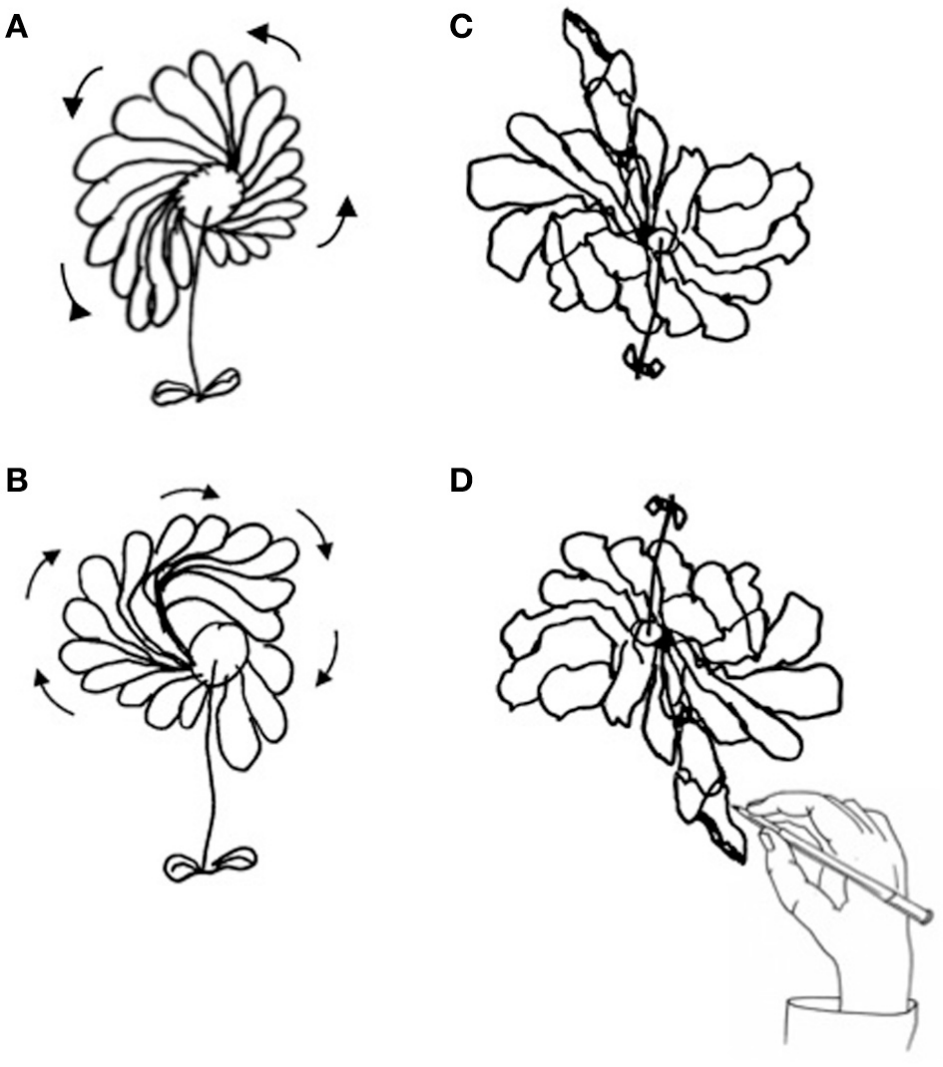
**Drawing of daisy from memory by P#1: in the clockwise direction (A), in counter-clockwise direction (B), and in the epidiascope condition [daisy seen (C), daisy drawn (D)]**.

**Figure 4:**
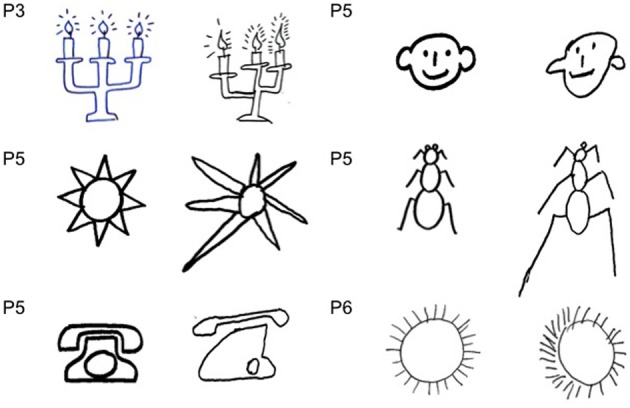
**Examples of drawing by copy (P#3: candle), (P#5: star, phone, face, ant), and (P#6: sun)**.

**Figure 5:**
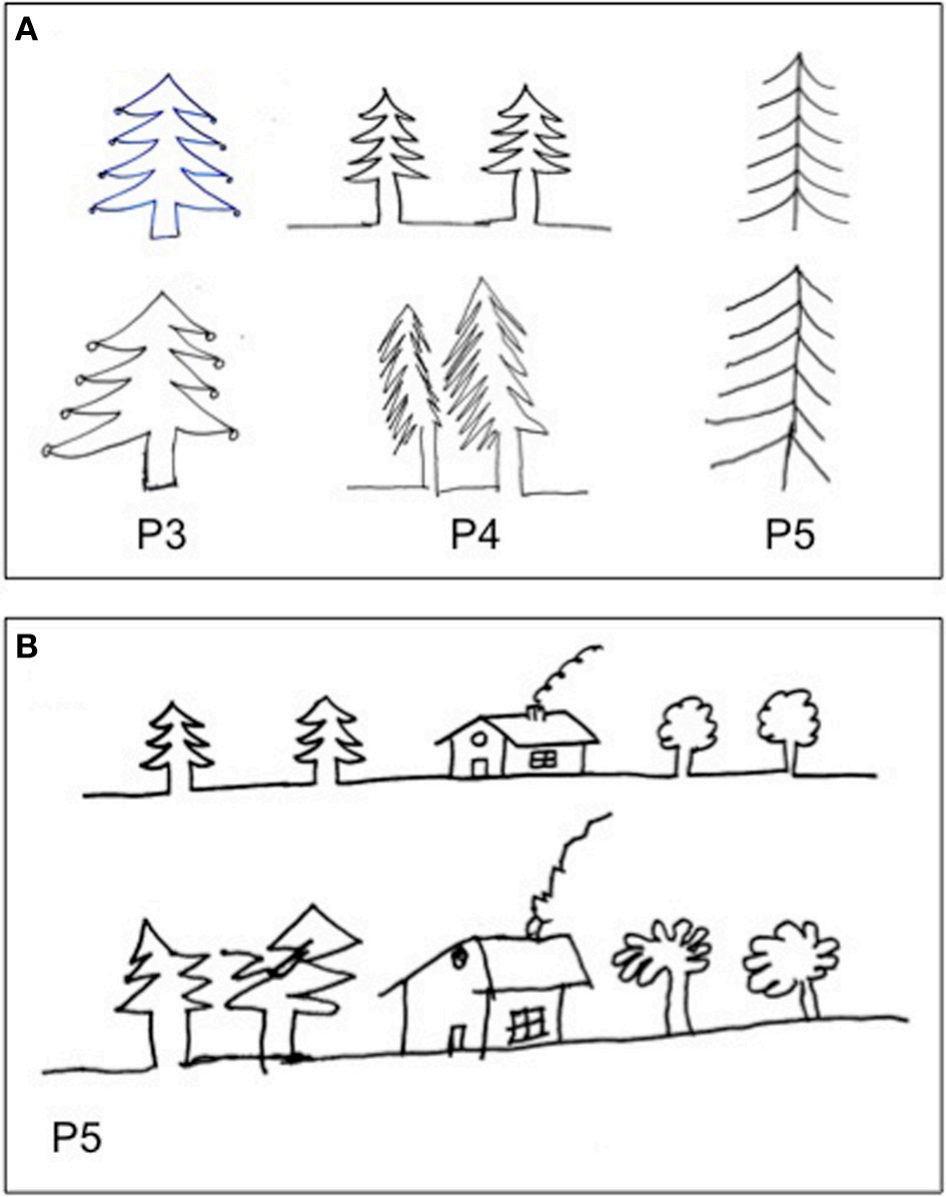
**Examples of drawing by copy (A, P#3, P#4, and P#5: fire tree), (B, P#5: house and trees (Gainotti et al., 1972).** The patients drew greater left-hand side of objects, even when objects were located to the right side of model **(B)**.

In drawing tasks all patients produced some putatively symmetrical objects (a house, a fir tree, a Christmas fir tree, a man, a butterfly, a daisy) disproportionately larger on the left side. This behavior was particularly evident in the case of the daisy. It was independent of the direction of the handwriting; indeed the same enlargement of the left side was observed when patients draw from memory a daisy in clockwise or counter clockwise direction. Throughout the investigation, it was repeatedly noted that patients were unaware of the left size distortion, that was denied at specific questioning by the examiner (Figures 2–5).

All patients gave their informed consent prior to the study. Control (C) data were provided by six right-handed neurologically unimpaired participants [three females; mean age: 54.8 years (range 34–78); mean years of schooling: 12 (range 9–18)] from previous studies (Rode et al., 2006, 2008).

## Experimental study

The patient's processing of leftward and rightward extent was assessed by the procedure used by Rode et al. (2006). For the quantitative analysis of patients' performance in the drawing tasks by copy and from memory, we used the daisy, which was given to all patients with the same procedure. Nevertheless, patients exhibited a disproportionate enlargement of the left side of figures in many other drawings (see some examples in Figures 2–5).

### Line bisection

The test devised by Schenkenberg et al. (1980) was used. The stimuli included 20 lines. Eighteen lines were organized in three sets of six lines, so that one set lay primarily on the left side of the page, one in the center, and one on the right side. Each set contained lines of 100, 120, 140, 160, 180, and 200 mm. The lines were organized so that the test was balanced with regard to line length from top to bottom. Moreover, two additional lines (150 mm), placed at the top and bottom (center), were used in communicating instructions to participants, and were also included in data analysis. Participants received instructions to mark the center of each line with a soft pen, without skipping any stimulus. All patients and controls performed the task, with their right hand, namely: the unaffected hand, ipsilateral to the side of the right hemispheric lesion in the seven brain-damaged patients. The length of the left side of the line (i.e., from the left end of the line to the subject's mark) was measured to the nearest mm. That measurement was converted to a standardized score (percent deviation), using the formula: measured left half minus objective half/objective half × 100. This transformation yields positive numbers for marks placed to the right of the center, negative numbers for marks placed to the left of the center.

### Drawing from memory

Participants were required to draw from memory a daisy, on a 21 × 29.7 cm sheet of paper, placed in front of them, with the center of the sheet aligned with the mid-sagittal plane of the patients' body. No model was provided, and no further specific instructions were given. In order to measure the area and the number of petals of the left and of the right sides, each drawn daisy was divided into two sides by a vertical line passing through the center of its pistil. Petals divided into approximately equal parts by the vertical line were not considered in counting the number of drawn petals. Occasionally, patients added leaves, which were not considered for the analyses. The areas of the two sides of the drawing were computed by a Leica imaging system and a Quantimet 500 software. For the left- and right-sided areas of each drawing, a laterality index score (LI) was computed: (left-sided area minus right-sided area/left-sided area plus right-sided area) × 100. A positive value of this LI indicates a greater left-sided area, a negative LI a greater right-sided area. A similar LI was computed for the number of petals (PLI). Each participant was required to draw six daisies. All seven patients showed no difficulties in performing the test, and drew the pistil and the right-sided petals first. Control participants drew the pistil first, and did not show any definite pattern as for the petals.

The role of input (perceptual) vs. output (premotor) factors (Vallar and Mancini, 2010) in bringing about the pathological enlargement of the left side of drawings was assessed by the epidiascope method (Nico, 1996), in P#1 and P#2. This procedure (Coslett et al., 1990) decouples the direction movement of the hand from the participant's visual control of the display. Patients with a putatively perceptual disorder would show a left-sided deficit (omissions in a cancellation task, a disproportionate enlargement of the left-hand side of the drawing in the present investigation) with reference to their field of vision (normal vs. mirror-reversed), independent of the direction of the movement of the arm and hand. Patients with a premotor disorder would exhibit a left-sided deficit, with reference to the mid-sagittal plane of the body, independent of the normal or mirror-reversed field of vision.

In P#1, the role of visual control was assessed by a blindfold condition (three trials were given). In this study, control data were collected from six neurologically unimpaired participants (three males; mean age 54.5 years, range 30–81 years), with three participants being different from those participating in the other experiments. Each control participant was given six trials.

### Drawing by copy

This test differed from the previous one in that a daisy symmetrical model was provided. The model was printed in the center of a 21 × 29.7 cm sheet. Six trials were given. The data were analyzed as in the drawing from memory study. The same participants who took part in the previous experiment executed also this task, except for P #2.

### Perceptual matching task

The stimuli were pairs of black rectangles 15 mm high, with a 6 cm empty space between them. Twenty-five pairs of rectangles were presented in a pseudorandom series, in order to measure the point of subjective equality between patterns placed in the left and right visual half-spaces. The distance between the right side of the left-sided rectangle and the left side of the right-sided rectangle was 8 cm. The center of this distance was aligned with the mid-sagittal plane of the participant's trunk. In each pair, the length of one rectangle was fixed (8 cm), the length of the other varied from 6.4 to 9.6 cm, in four 8 mm steps. In five trials the two rectangles were equal in length (8 cm). In ten trials the right-sided rectangle was longer than the left-sided segment, in ten trials vice-versa. The participant's task on each trial was to report verbally which was the longer out of the two rectangles. The scoring procedure of Milner et al. (1993) was used. Each error on a given trial was scored a value of *n* ± 1, where *n* was the number of steps by which the patterns' lengths differed on that trial. Rightward errors (i.e., the right-sided rectangle judged as longer, when the left-sided rectangle was longer), were given a positive score. Leftward errors (i.e., the left-sided rectangle judged as longer, when the right-sided rectangle was longer) were given a negative score. Using this scoring method, an identical pair of stimuli (*n* = 0) yielded a score of either +1 (rightward error) or −1 (leftward error). The larger was the difference in length between the two rectangles, the greater the error score. All seven patients and six neurologically unimpaired control participants were evaluated in this task.

### Line extension task

The participants' task was to reproduce the length of a horizontal line in two conditions. In the rightward movement condition, the right end of the line was aligned with the mid-sagittal plane of the body, and the patient reproduced the perceived length of the segment with a rightward extension. In the leftward movement condition, the left end of the line was aligned with the mid-sagittal plane of the body, and the patient made a leftward extension. The stimuli were horizontal black lines, 1 mm in width, with three line lengths (4, 6, and 8 cm). Twenty-four lines (eight for each length) were given to each participant, in a random fixed order, with the exception of P#1, who extended 18 lines. In other patients and control participants, 24 lines were presented. The length of the segment drawn by each participant on each trial was measured to the nearest mm. For the leftward extension of each line drawing, the following laterality index score (LI) was computed: Leftward extended length minus length of the right-sided line/leftward extended length plus length of the right-sided line × 100. For the rightward extension of each line drawing, the LI was: Rightward extended length minus length of the left-sided line/rightward extended length plus length of the left-sided line × 100. Positive/negative values of the LI indicate over/under-extension. Mean LIs were computed for the two directions of extension (rightward, leftward), and for three line lengths (4, 6, and 8 cm). All seven patients and six controls were tested.

## Statistical analyses

The line bisection, drawing, and perceptual matching tasks were analyzed by non-parametric statistics (Siegel and Castellan, 1988), comparing the performances of patients and neurologically unimpaired participants. The individual patients' scores were compared with the mean score of control group by *t*-tests (Crawford and Howell, 1998; Crawford and Garthwaite, 2002). For the line extension task, the scores obtained by the two groups in left/right extension of lines were analyzed using an ANOVA. However, rather than making use of the standard cumulative distribution function (as in standard ANOVAs), we re-estimated the null-distribution for each effect through 5000 bootstrap resamples with replacement from our original dataset (Berkovits et al., 2000; Wilcox, 2012). This approach allows robust inferences from factorial designs even under conditions (e.g., limited number of participants), where assumptions for using parametric distributions are not met. Non-parametric Spearman correlation analyses were performed to explore the correlation between the performance of patients in tasks assessing spatial functions (line bisection) and HS.

## Results

### Line bisection

Patients (*N* = 7) and control participants (*N* = 6) had an average percent negative score, indicating a leftward deviation in both groups (patients: −2.21, *SD*: 4.82; controls −0.71, *SD*: 2.46), with no significant difference on a Mann–Whitney test (*z* = −0.86, *p* =n.s.).

### Drawing from memory and by copy

**Figure 6:**
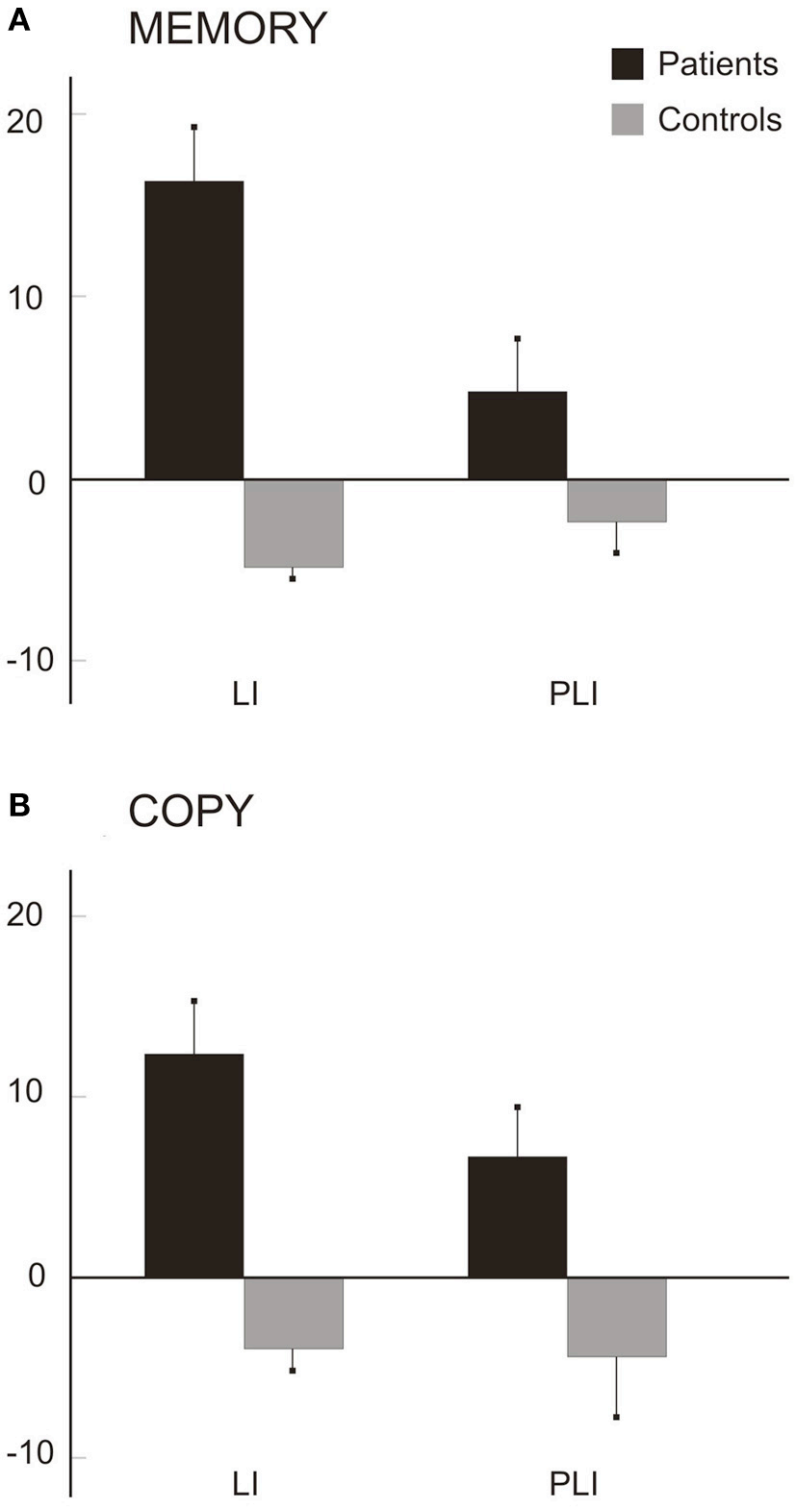
**Scores for daisy drawings from memory (A) and by copy (B).** Mean LIs (s.e.m.) for drawn areas and mean PLIs (s.e.m.) for number of petals in the seven patients and six control participants.

Figure 6 shows the average scores (from memory: A; by copy: B). Patients' scores were greater and positive in both indexes, indicative of a greater left-sided drawn area (LI) and of a greater number of left-sided petals (PLI) drawn; on the contrary, the mean LI and PLI scores of control participants were slightly negative.

In the daisy drawings from memory, Mann–Whitney tests revealed significant differences between the two groups for LI (*z* = 2.93, *p* = 0.003) but not for PLI (*z* = 1.64, *p* = 0.100) scores. A perusal of the memory data shows a greater difference for LI (*N* = 7 for patients, *N* = 6 for controls; Patients: mean LI = 16.30, *SD*: 7.81; C: mean LI = −4.87, *SD*: 1.34) but only a trend for PLI (Patients: mean PLI = 4.78, *SD*: 7.64; C: mean PLI = −2.39, *SD*: 4.04) scores between groups. In the daisy by copy, Mann-Whitney tests revealed that LI (*z* = 2.64, *p* = 0.008) and PLI (*z* =2.02, *p* = 0.044) scores are greater in patients (*N* = 6; mean LI = 12.34, *SD*: 7.3; mean PLI = 6.70, *SD*: 7) than in control participants (*N* = 6; mean LI = −3.94, *SD*: 3.18; mean PLI = −4.43, *SD*: 8.17).

In the epidiascope condition, P#1 and #2 drew a larger left-hand side of the daisy with reference to their field of vision. Indeed, the direction of the hand movement was preferentially oriented toward the right side (see Figures 3C,D). The LIs, computed on the seen drawing, were +24.4 for P#1 and +8.7 for P#2. Values of LI for number of petals were respectively, +22.2 for P#1 and +11 for P#2.

In the blindfold condition, without visual control, P#1 drew the left-hand side of the daisy much larger than the right-hand side, on all three trials: the LI score was +56.0 (range: +32.3 to +79.2). The average LI score was of six control participants was −8.72 (*SD*: 4.71; range: −2.66 to −14.85). P#1 LI score was compared with the average control data by a *t*-test (single patient analysis, see Crawford and Garthwaite, 2002), showing a difference (*t* = 12.72, *p* < 0.001). The PLI score was +40.0 (range: +14.2 to 53.8). The average PLI score of six control participants was −8.19 (*SD*: 3.62; range: −4.32 to −14.10). A *t*-test comparing the PLI score of the patient with the control data, showed a significant difference (*t* = 12.32, *p* < 0.001).

### Perceptual matching task

Both patients (*N* = 7) and controls (*N* = 6) had positive scores (1.098; *SD*: 0.28; 0.905; *SD*: 0.11), marginally greater for the patients' group, suggestive of a rightward bias, with some perceptual underestimation of left extent, with a trend toward significance (Mann-Whitney test: *z* = 1.79, *p* = 0.074). Based on the trend resulting from the analysis and on the findings previously found in the first three patients (see Rode et al., 2006, 2008), we compared each patient's score with the controls' mean score by *t*-tests [single case analysis, see Crawford and Garthwaite (2002)]. The scores of P#1 (1.31, *t* = 3.41, *p* = 0.019), P#6 (1.33, *t* = 3.58, *p* = 0.016), and P#7 (1.23, *t* = 2.74, *p* = 0.041) were significantly different from the controls' mean score; the difference between P#2's score (1.19) and the control score approached the significance level (*t* = 2.399, *p* = 0.062). The scores of P#3 (1.14) and P#5 (0.95) did not differ from the controls' average score. Finally, the score of P#4 (0.53) indicates a significantly minor rightward bias, as compared to that of controls (*t* = −3.16, *p* = 0.025). In conclusion, While four patients show rightward bias greater that than the average of controls, this is not the case for the other three patients, with one actually scoring in a significantly opposite direction.

### Line extension task

**Figure 7:**
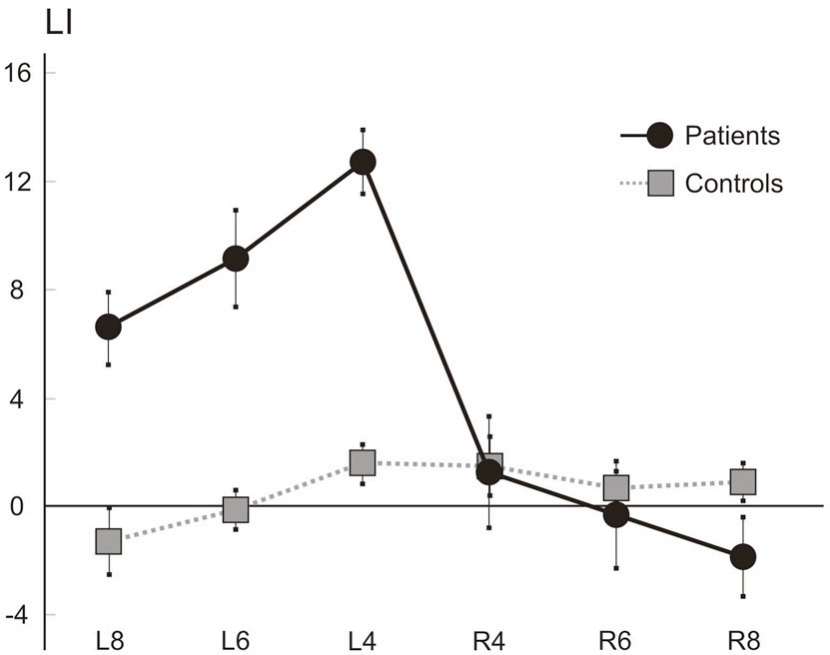
**Line extension task.** Mean (s.e.m.) LIs of the seven right brain patients and six control participants for rightward (R) and leftward (L) extended lines (length: 4, 6, and 8 cm).

Figure 7 shows the average percent LI scores of patients (*N* = 7) and control (*N* = 6) participants. On average, patients showed a leftward overextension, while the laterality scores of patients for rightward extension appeared broadly comparable to those of control participants, with only a mild rightward hypoextension in the longest lines (8 cm). A factorial ANOVA with Laterality (2 levels: Left and Right) and Length (3 levels: 4, 6, and 8 cm) as the within-subjects factors, and with Group (2 levels: patients and controls) as the between-subjects factor, was performed. The F distribution was re-estimated via 5000 bootstrap resamples of our dataset (Berkovits et al., 2000). The main factors of Group (*F* = 12.96, estimated bootstrap *p* = 0.003), of Laterality (*F* = 37.21, estimated bootstrap *p* < 0.001), and the Laterality by Group interaction (*F* = 45.71, estimated bootstrap *p* < 0.001). were significant. Paired comparison tests revealed that patients and controls differed for left (patients' mean score: 9.48, *SD*: 3.16; C mean score: 0.08, *SD*: 1.64; *p* < 0.001) but not right (patients' mean score: −0.26, *SD*: 2.97; C mean score: 1.03, *SD*: 1.28; p = n.s.) extension LI scores, with patients obtaining greater and positive LI in leftward extension, indicating overextension. Finally the main effect of Length (*F* = 6.28, *p* < 0.001) was significant: paired comparison tests indicated that, on average, the short lines (4 cm; mean score: 4.5, *SD*: 6.13) presented with greater scores (and therefore overextension) with respect to the medium (6 cm, mean score: 2.5, *SD*: 5.46; *p* < 0.001) and the long (8 cm, mean score: 1.2, *SD*: 4.65; *p* < 0.01) ones. No other factor was significant.

### Correlation analyses

Spearman correlation analyses between the scores in the tasks assessing different features of HS (i.e., LI and PLI scores in drawing by copy and from memory; score in the perceptual matching task; LI score for leftward line extension), and the scores in a task assessing spatial functions (i.e., line bisection), showed no significant correlations in any of the measures considered (all *p* > 0.17).

## Discussion

Four right-brain-damaged patients with left spatial HS are reported, and three right-brain-patients, previously studied (Rode et al., 2006, 2008) are reviewed, for a total of a series of seven patients. The HS productive (Vallar, 1998) symptom-complex consists in the leftward overexpansion of drawn objects and in the leftward overextension of lines. HS patients are not aware of this leftward disproportionate expansion. The perceptual underestimation of the extent of left-sided objects, originally found by Rode et al. (2006, 2008) was not shown in some novel patients, then undermining the role of this deficit in bringing about HS in drawing. At least in patients who do not perceptually underestimate left extent, left HS (namely, leftward expansion in drawing) is unlikely to reflect a compensatory response aiming at making symmetric objects which are perceived as less extended on the left.

A limitation of this study is that we did not include a control group of neurological patients with brain lesions but without HS. Future investigations may assess this issue, comparing the performance of right-brain-damaged patients with HS with those of other neurological groups, such as right- and left-brain-damaged patients without contralesional neglect and HS, and right-brain-damaged patients with different subtypes of spatial neglect (such as, for instance egocentric vs. allocentric, see Vallar and Bolognini, in press). With these caveats in mind, the data from the current series of seven patients, as compared with a group of neurologically unimpaired control participants are, nevertheless, informative about the key features of HS, confirming the findings of Rode et al. (2006).

The left-sided distortion of size in drawing tasks from memory or by copy is influenced neither by the direction of the movement of the hand nor by visual control, suggesting that a disorder of motor programming and action execution toward the left side of the space cannot explain this disorder. It takes place in the left visual field, independent of the direction of the movement of the upper limb, manipulated through a mirror-reversed vision of action. Five out of seven patients had a left homonymous hemianopia and two no visual deficits, suggesting that the disorder is not modified by the presence or absence of a visual primary deficit. Moreover, left spatial HS remained unchanged when patients were asked to draw in a blindfold condition. All these findings are in favor of a mainly spatial, rather than purely visual, nature of the deficit. The spatial nature of HS is also suggested by the presence of disorder in 3D (modeling), visuo-constructional tasks, where the proprioceptive-somatosensory inputs play a relevant role in the spatial disorder (Rode et al., 2008).

The spatial higher-order reference frame of HS differs from the sensory visual frame nature of hemimicropsia, a rare disorder of visual perception characterized by a reduction of the perceived size of objects in one visual half-hemifield (Cohen et al., 1994; Frassinetti et al., 1999; Kassubek et al., 1999). Patients with hemimicropsia report seeing objects in the contralesional hemifield smaller in size, as compared with objects in the ipsilesional hemifield. Unlike patients with spatial HS, hemimicropsic patients are aware of their disorder and tend to compensate for their deficit: they draw the contralesional side of objects larger than the ipsilesional side and correct symmetric patterns, without addition of details; the disorder is only confined to vision. In conclusion, on the basis of this pattern, hemimicropsia may be considered as a modality-specific disorder of visual size perception, in retinotopic reference frames, consecutive to damage to the visual association cortex (extra-striate association visual cortex, BAs 18 and 19: see Cohen et al., 1994; Frassinetti et al., 1999; Kassubek et al., 1999). Conversely, spatial HS may be considered as a non-modality-specific distortion space representation (Rode et al., 2006, 2008).

The three right-brain-damaged patients described by Critchley (1953) and the one reported by Guariglia et al. (1998) also exhibit a left visual neglect, asking about the possible relationship between these two disorders. In the present study, all patients had shown evidence for left spatial neglect during an early period post stroke onset, but these symptoms had disappeared at the time of examination. It could be also possible that the patients' behavior (drawing a larger left-hand side of objects, leftward overextension) reflects a compensatory disproportionate attention toward the contralesional side of the space during recovery from left spatial neglect. However, in the seven patients no behavioral bias was evidenced in different tasks, including line bisection (Schenkenberg et al., 1980). Furthermore, a compensatory behavior does not explain the underestimation of the size of left-sided objects (Rode et al., 2006), which, if anything may indicate a left perceptual neglect. Furthermore, as noted above, one novel result from the present series is that left perceptual underestimation of extent was not found in all HS patients. Consequently, left spatial HS cannot be explained in terms of a general unilateral spatial neglect affecting the left side of space, but it has to be considered as a distinct spatial disorder, which could nevertheless co-occur with neglect following right brain damage (Halligan et al., 2003; Heilman et al., 2003; Vallar and Bolognini, in press).

The patients' performance in drawing from memory and by copy suggests that the derangement of different spatial processes brings about left neglect and HS. On the one hand, right-brain-damaged patients with left neglect omit left-sided elements of objects, suggesting a deficit (i.e., such as a restriction) of the internal representation of the contralesional side of space, with the possible co-occurrence of ipsilesional perseveration (Rusconi et al., 2002; Nys et al., 2006; Ronchi et al., 2009, 2012). On the other hand, in patients with HS drawings reveal a different type of misrepresentation (expansion) of contralesional space, without modification of the right side of the object. A further difference between neglect and HS concerns the presence of perseveration behavior. Right-brain-damaged-patients with spatial neglect, particularly when the lesion involves frontal, insular and subcortical regions, may show pathological manifestations mainly affecting the ipsilesional right-hand side of space, ranging from the repeated cancelling out of targets to the addition of new targets and/or more complex graphic productions (Na et al., 1999; Manly et al., 2002; Rusconi et al., 2002). This perseveration phenomenon differs from the productive symptoms of patients with HS. At first, in HS, productive symptoms concern the contralesional, rather than ipsilesional hand-side of space, with patients adding more details on drawings as, for example, more petals on a daisy, a supplementary left wing or more eyespots within the left wing of butterfly (Figure 2). Secondly, unlike perseveration behavior of patients with spatial neglect, patients with HS do not make perseverative marks in target cancellation tasks, or other graphic additions: even though sometimes right-brain-damaged patients without perseveration in target cancellation tasks exhibit perseveration in drawing tasks, this productive symptom affects the right-hand side of space, with addition of irrelevant elements, and with no leftward contralesional expansion of drawings (Ronchi et al., 2009). Notwithstanding these differences, unawareness of the deficit is a hallmark of both spatial disorders (neglect and HS), suggesting that HS, as spatial neglect, includes a monitoring component.

In HS, the leftward contralesional expansion in drawing by copy may suggest some influence of the perceived visual stimulus, as for visual illusions of length and displacement (Ro and Rafal, 1996; Vallar et al., 2000). However, the presence of the disorder also when no visual information is available, such as in drawing from memory, and in blindfold conditions, indicates that the leftward enlargement or displacement is not specific for sensory modality, concerning instead spatial frames or medium (Vallar, 2009). We suggested that the disproportionate enlargement of the left-hand side of objects may be due to a lateral anisometry of the internal representation of space, namely: the spatial medium with a leftward relaxation (Rode et al., 2006, 2008). Based on this account, two predictions could be made: left-sided stimuli could be perceived as smaller than right-sided stimuli, with a resulting left overextension in line extension. In the perceptual matching task, the comparison of the performances of patients and controls indicates a trend toward significance. However, the analysis of the individual score of patients shows that only four out of seven HS patients (P#1, P#2, P#6, P#7) presented with a rightward overestimation; two other patients (P#3 and P#5) performed as the control group of neurologically unimpaired participants, and one patient (P#4) presented with an opposite pattern, namely a leftward overestimation. These findings suggest that a perceptual disorder cannot account for HS, being possibly instead an associated deficit with no direct causal relationships with HS in drawing, namely the main clinical manifestation of the deficit. In line with these findings–as to the lesion correlates of HS—while HS patients showing a perceptual rightward bias had lesions involving the frontal and insular cortex and subcortical areas (head of caudate nucleus, putamen, internal capsule, white matters under frontal lobe), the two patients scoring in the normal range had these brain structures intact. The limited number of patients calls for caution as for the anatomical implications of the present findings. Nevertheless, the conclusion can be drawn that a perceptual rightward bias is not a core mechanism of left HS, and there is some indications that its neural underpinnings may be different at least in part.

In the line extension task, involving not only an estimation of the lateral extent/length of the line, but also the programming, monitoring and execution of a lateral movement, HS patients make a leftward overextension (see Figure 5), while laterality scores for rightward extension do not differ from those of controls. These findings, together with the observation that a perceptual underestimation of left-sided extent is not a deficit consistently found in all patients, suggest that HS emerges when operations (including planning and execution of motor acts in the left side of space) are required.

**Figure 8:**
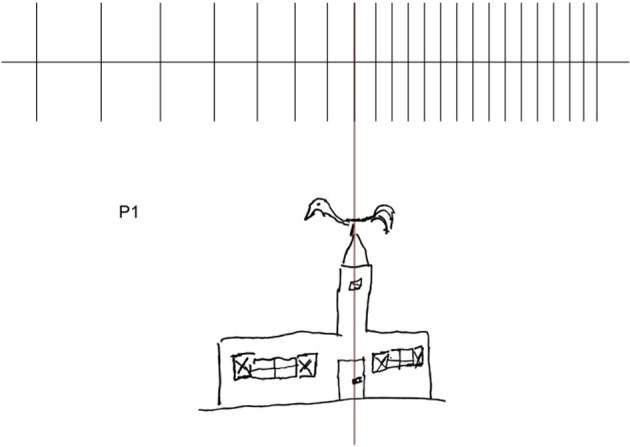
**Example of drawing from memory (P#1: church)**.

This leftward expansion of spatial representations may be illustrated by the drawing of a church made by P#1: the disproportionate enlargement concerns the left-hand side of the church, the windows, particularly the shutters, the door and the beak of rooster bell (see also Rode et al., 2006, 2008) (see Figure 8).

This leftward expansion of the spatial medium affects the size representation of extra-personal space, along the horizontal dimension according to an object-centered frame. A similar HS due to a right-sided frontal vascular lesion has been reported by Saj et al. (2011), in a right-handed patient with no spatial neglect or hemianopia. As for the seven patients of the study, drawings from memory revealed a disproportionate expansion and addition of details to the object (a flower, an unfolded cube), reflecting an expansion of the spatial medium but, in Saj's patient, ipsilateral to the side of the lesion (Ipsilateral HS).

More generally, a disproportionate enlargement of space representation may also affect bodily space. The first description has been made by the French otolaryngologist Bonnier (1905), based on the clinical observation of patients with vestibular disorders of peripheral origin [for an historical note see Vallar and Rode (2009): see also Bonnier's book “Le vertige” (Bonnier, 1904)]. Bonnier reported that these patients may subjectively feel that their body or body-parts are disproportionately enlarged, and interpreted this disorder as a pathologic expansion of the spatial representation of the body or body parts (HS) (for an historical discussion of the terms “schema” and “hyperschematia” see Vallar and Papagno, 2003). These feelings also resemble those reported by patients with macrosomatognosia or somatoagnosic hallucinations, with patients referring the feeling that one or more parts of their body are disproportionately larger (Lhermitte and Tchehrazi, 1937; Frederiks, 1969; Denes, 1989), after paroxysmal cerebral disorders such as epilepsy, migraine and hypnagogic hallucinations (Kew et al., 1998; Podoll and Robinson, 2002), somatosensory loss consecutive to a local anaesthesia (Gandevia and Phegan, 1999; Paqueron et al., 2003), or a brainstem lesion (Rode et al., 2012). But unlike HS for extra-personal space, patients are always aware of their deficit, suggesting different underlying neural and functional mechanisms and neural processes as for disorders affecting the representation of the size (and the volume) of a portion of extra-personal or personal spaces.

In sum, HS for left extra-personal space is a rare neuropsychological deficit brought about by right brain damage. Its main characteristics include: leftward expansion in drawing both by copy and from memory (as shown in one patient, leftward expansion in modeling); overextension in leftward line extension, while rightward line extension is within the normal range. Signs of left spatial neglect are not a feature of HS, including right perceptual overestimation of extent, as well as left hemianopia. HS is most frequently contralateral to the right hemispheric lesion, as left spatial neglect. As for ipsilesional (right) neglect (Kwon and Heilman, 1991; Adair et al., 1998), however, a few reports of ipsilesional right HS (Saj et al., 2011), and of ipsilesional right overextension (Bisiach et al., 1998) are on record.

### Conflict of interest statement

The authors declare that the research was conducted in the absence of any commercial or financial relationships that could be construed as a potential conflict of interest.
